# Supplementary material for: Association between oral microbiome and seven types of cancers in East Asian population: a two-sample Mendelian randomization analysis
Source: Front Mol Biosci. 2023 Nov 21;10:1327893. doi: 10.3389/fmolb.2023.1327893 (PMC10702768; doi:10.3389/fmolb.2023.1327893)
Supplement: Supplementary file 1 [file DataSheet1.ZIP › Supplementary Materials/MR plots for tongue/tongue═╝/Ovarian cancer/pheno.2607_to_ovarian cancer_forest.pdf]

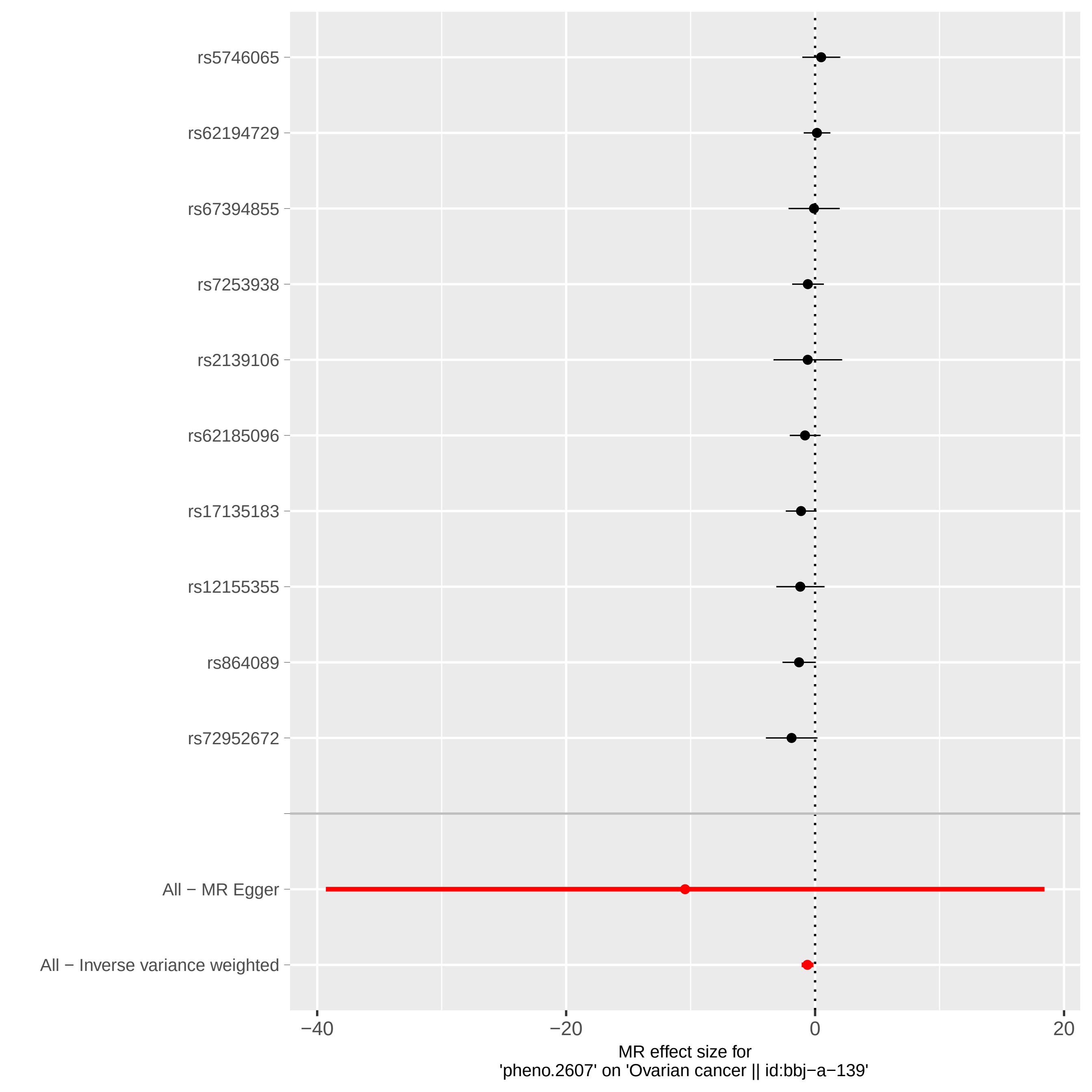
rs5746065
rs62194729
rs67394855
rs7253938
rs2139106
rs62185096
rs17135183
rs12155355
rs864089
rs72952672
All − MR Egger
All − Inverse variance weighted
−40
−20
0
20
MR effect size for
'pheno.2607' on 'Ovarian cancer || id:bbj−a−139'
